# Genotype-by-Environment Interactions and Response to Selection for Milk Production Traits in Lacaune Sheep from Greece and France

**DOI:** 10.3390/vetsci12030194

**Published:** 2025-02-21

**Authors:** Sotiria Vouraki, Jean-Michel Astruc, Gilles Lagriffoul, Rachel Rupp, Georgios Banos, Georgios Arsenos

**Affiliations:** 1 Laboratory of Animal Production and Environmental Protection, School of Veterinary Medicine, Faculty of Health Sciences, Aristotle University, 54124 Thessaloniki, Greece; svouraki@uoi.gr; 2 Laboratory of Animal Production, Nutrition and Biotechnology, Department of Agriculture, School of Agriculture, University of Ioannina, Kostakioi, 47100 Arta, Greece; 3 Institut de l’Elevage, CS52637, 31321 Castanet-Tolosan, France; jean-michel.astruc@idele.fr (J.-M.A.); gilles.lagriffoul@idele.fr (G.L.); 4 INRAE, INPT-ENVT, INPT-ENSAT, GenPhySE, 31326 Castanet-Tolosan, France; rachel.rupp@inrae.fr; 5 Scotland’s Rural College (SRUC), University of Edinburgh, Midlothian EH25 9RG, UK; georgios.banos@sruc.ac.uk

**Keywords:** Lacaune sheep, genotype, environment, genetic gain, milk production

## Abstract

The objective of the study was two-fold and is as follows: (i) to investigate genotype-by-environment (G × E) interactions for milk yield and composition in purebred Lacaune sheep reared intensively in Greece and semi-extensively in France, and (ii) to estimate the potential genetic gain of selection based on genetic evaluations jointly calculated in both countries compared to each country separately. A total of 1658 Lacaune ewes from 4 intensive farms in Northern Greece and 4859 Lacaune ewes from 186 semi-extensive farms in Southern France were used. Ewes in the two countries were daughters or granddaughters of the same rams (6 common sires and 11 common grandsires). Individual ewe milk yield was recorded monthly, and milk samples were collected to assess their chemical composition. The total milk yield and milk component content were calculated. Results showed a strong genetic correlation for milk yield (0.86 ± 0.13, *p* < 0.05) and protein content (0.88 ± 0.12, *p* < 0.05) between the two countries, suggesting no strong evidence of G × E interaction. In the case of fat content, a moderate correlation was found (0.59 ± 0.21, *p* < 0.05) indicating some degree of sire re-ranking. Results suggest that a joint genetic evaluation and selection of Lacaune sheep in Greece and France is feasible. Simulations showed that selection across countries is estimated to increase genetic gain up to 68.17% and 0.16% in Greece and France, respectively, as compared to selection within a country.

## Simple Summary

Improving milk production traits of small ruminants is a desirable breeding goal to maximize farm profitability. Genetic selection for these traits using data from different countries could increase progress and benefit breeding programs, particularly in small ruminant production systems that are characterized by great diversity across countries. Therefore, a study was undertaken within the SMARTER project to investigate the feasibility of genetic evaluation and selection for milk yield and composition in purebred Lacaune sheep reared intensively in Greece (*n* = 1658) and semi-extensively in France (*n* = 4859). The results showed a strong genetic correlation between milk yield and protein content and a relatively high correlation between fat content and animals raised in the two different countries. Consequently, there is no evidence of genotype-by-environment interaction. This suggests that a joint genetic evaluation of Lacaune sheep in Greece and France is feasible. Joint genetic evaluation and selection are expected to increase genetic gain in both countries.

## 1. Introduction

Dairy sheep farming is a significant agricultural activity in Mediterranean countries, providing income and employment opportunities in remote areas [1]. In these countries, improving the milk production of dairy sheep is a desirable breeding goal to maximize farm profitability. Moreover, according to the latest research findings, milk yield and composition could be used as proxy traits of feed efficiency in dairy sheep [2,3]. Therefore, selection to increase milk production could also lead to improved feed efficiency. The latter could help further increase farm economic performance and reduce environmental impact.

Genetic selection for milk production traits using and combining data from purebred animals of the same breed, reared under different conditions (countries, farming systems, climates), could enhance progress and benefit breeding programs. Specifically, such an approach would increase the number of selected candidates, thus resulting in a higher selection intensity and genetic gain [4,5].

However, dairy sheep farming is characterized by great diversity. Farming systems vary from fully extensive to intensive ones, characterized by different management and feeding practices. Moreover, available feed resources, their quality, and climatic conditions vary by geographical region [6]. Therefore, to implement successful breeding programs to improve milk production traits, it is important to investigate whether differences in selection responses should be expected depending on animal rearing and environmental conditions [7]. The latter is defined as genotype-by-environment (G × E) interactions.

Several studies have investigated G × E interactions for milk production traits in dairy cattle [8,9,10,11,12,13,14,15,16]. Many of these studies focused on animals reared in different countries and reported high genetic correlations (>0.80) for the studied traits, suggesting no or limited extent of G × E. However, relevant literature in dairy sheep is scarce [17,18,19]. Moreover, to the best of our knowledge, there are no available studies investigating the potential genetic gain of selection by combining genetic evaluations from dairy sheep reared under different conditions.

Sheep populations in Greece and France are among the largest in Europe, with 7.3 million and 6.6 million animals reared, respectively [20]. In Greece, this population consists entirely of dairy sheep breeds (indigenous and foreign), which are primarily reared under intensive or semi-intensive conditions [21]. In France, one-third of the sheep population is also primarily used for milk production, including mainly indigenous breeds reared under semi-extensive conditions [1]. Among these breeds is the highly productive Lacaune breed, which has also been largely imported in Greece during the past decade.

Therefore, the objective of the present study was two-fold and is as follows: (i) to investigate G × E interactions for milk yield and composition traits in purebred Lacaune sheep reared in Greece and France, and (ii) to estimate the potential genetic gain of selection based on genetic evaluations jointly calculated in both countries compared to within each country separately.

## 2. Materials and Methods

### 2.1. Ethics Statement

The study was approved by the Research and Ethics Committee, School of Veterinary Medicine, Faculty of Health Sciences, Aristotle University of Thessaloniki, Greece (H813/09.01.2019). The study was conducted in accordance with the local legislation and institutional requirements.

### 2.2. Animals and Farms

**Figure 1:**
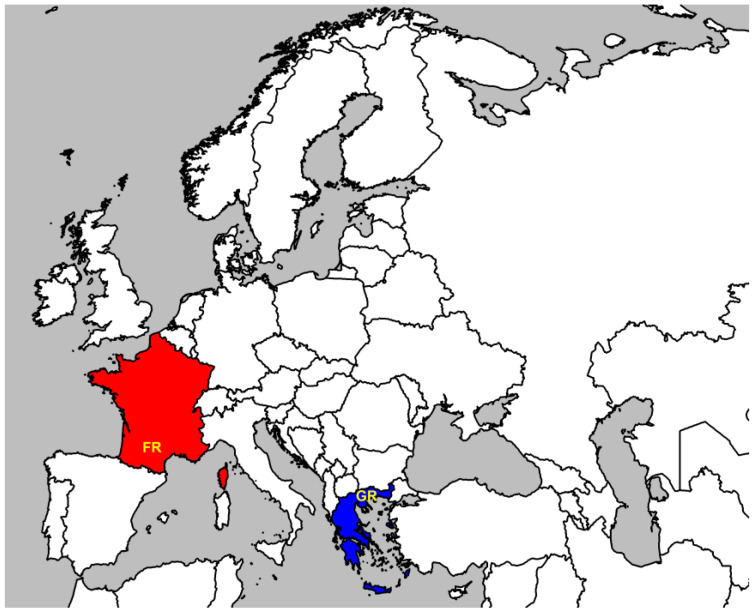
Map illustrating the regions in Greece (GR; mean latitude 41.21394575 and longitude 24.01874196) and France (FR; mean latitude 44.0803165 and longitude 2.731293321) where the studied farms were located.

A total of 2000 Lacaune ewes from four intensive farms in Northern Greece (mean latitude 41.21394575 and longitude 24.01874196) and 4859 Lacaune ewes from 186 semi-extensive farms in Southern France (mean latitude 44.0803165 and longitude 2.731293321) were selected for the study (Figure 1).

Ewes in Greece were all born after artificial insemination (AI), using semen of imported Lacaune rams (*n* = 14) from France. During the study period, all ewes were in their first or second parity and were fed a total mixed ration consisting of alfalfa silage, alfalfa hay, and a high level of concentrates. Ewes in France were selected to be genetically related to those in Greece through 6 common sires and 11 common grandsires and were in their first to seventh parity during the study period. They mainly grazed on pastures and were also supplemented with concentrates and forages. All studied French farms reared at least five ewes that were genetically related to ewes in Greece.

A combined pedigree file was created for the Greek and French populations, including 26,547 animals, 2738 sires, and 17,361 dams (Dataset S1).

### 2.3. Phenotypic Data Collection and Editing

In Greece, individual animal recording was performed during the milking periods of years 2021 and 2022; 548 and 1452 ewes were monitored, respectively. Monitoring each animal started after lamb weaning (approximately 35 days post-partum) and lasted five months. Individual ewe milk yield was recorded monthly (5 records per animal) with volumetric milk meters. Moreover, milk samples (3 monthly samples per animal in early lactation) were collected in 50 mL tubes to assess their chemical composition—fat, protein, lactose, and solids-non-fat (SNF) content. Milk samples were transported to the laboratory at 4 °C, and their composition was determined with Near-Infrared Spectroscopy using a DA 7250 NIR analyzer (PerkinElmer, Waltham, MA, USA).

Individual-animal 24 h milk yield was calculated according to the official AT method of the International Committee of Animal Recording (ICAR) [22]. Then, total milk yield per milking period was calculated using the Fleishmann method of ICAR [22]. A minimum of three valid monthly records (>0.2 kg of milk) for each ewe was required to calculate milk yield per milking period, reducing the number of Greek ewes included in the study to 1658. The content of milk components was estimated as the arithmetic mean of individual monthly records weighted for milk yield. Quality control of milk composition records was implemented based on biological limits set for each trait (3–9%, 3–6.5%, 3–6.5%, and 10–15% for fat, protein, lactose, and SNF content, respectively). These limits set 3–7% of fat, protein, lactose, and SNF content records as missing values.

In France, a total of 7166 milking period records corresponding to years 2019, 2020, 2021, and 2022 from 1670, 1521, 1900, and 499 ewes, respectively, were obtained from the national genetic database (CTIG, Centre de Traitement de l’Information Génétique, Paris). Records included milk yield during the milking-only period and the fat content and protein content, which had been calculated from individual animal monthly records (five and three records for milk yield and content of milk components, respectively), as described in the case of Greece and following ICAR rules [22]. Fat and protein contents were in all cases estimated from milk samples collected during morning milking (AC method of ICAR).

Based on the above, the combined dataset from the two countries included 6517 ewes and 8822 milking period records. Additional data regarding age at lambing, the length of the milking period, and days from lambing to first sampling in both countries were available. The number of lambs born from each ewe was also available in France. Finally, the combination of monthly records used to calculate the average content of milk components for each milking period was defined as a categorical variable.

A description of the final datasets from Greece and France and the relationship of the studied phenotypes with other variables are presented in Table 1 and (Supplement S1 Figures S1–S12), respectively. The combined dataset from the two countries used for the analyses is presented in Dataset S2.

### 2.4. Data Analyses

#### 2.4.1. Within-Country Genetic Parameter Estimation 

Data from Greece and France were considered in a series of statistical analyses. Descriptive statistics of the studied animal phenotypes were derived using R statistical package “psych” [23]. Mixed linear models were used to identify environmental factors with significant effects on the studied animal traits in each country with R statistical package “lme4” [24]. Specifically, the fixed effects of farm, year, parity, age at lambing, number of lambs born, milking period length, days from lambing to first sampling, and combination of monthly records used to calculate the weighted average of milk component content were tested.

Within-country variance components of milk production traits were estimated in a series of univariate analyses using ASReml software version 4.2 [25,26]. Trait heritability and repeatability were calculated based on the corresponding variance values after convergence. The following model was used to analyze milk yield:

(1) $$Y_{\mathrm{ijklm}}=\mu +F_{i}+Y_{j}+P_{k}+b1\;\times M+b2\;\times A+b3\;\times D+L_{l}+A_{m}+e_{\mathrm{ijklm}}$$

where Y_ijklm_ = milk yield of animal m; μ = overall population mean; F_i_ = fixed effect of farm (4 levels in Greece and 186 levels in France); Y_j_ = fixed effect of year (2 levels in Greece and 4 levels in France); P_k_ = fixed effect of parity (2 levels in Greece and 7 levels in France); b1 = regression coefficient on milking period length M (days); b2 = regression coefficient on age at lambing A (months); b3 = regression coefficient on days from lambing to first milk record D (days); L_l_ = fixed effect of number of lambs born (2 levels); A_m_ = random additive genetic effect of animal m; and e_ijklm_ = random residual effect.

In the case of France, the random permanent environmental effect was also fitted in the above model to account for repeated records. For the within-country analysis of milk component content, the same model was used after including the fixed effect of the combination of monthly records used to calculate the weighted average of the milking period (11 levels).

#### 2.4.2. Genetic Correlations Between Countries—G × E

Estimated breeding values (EBVs) of common sires with daughters and/or granddaughters in both countries were derived directly from animal solutions in the above analyses for each country and trait, and their reliability was calculated using the following formula [27]:

(2) $$\mathrm{Rel}=1-\frac{\mathrm{PEV}}{\sigma_{A}^{2}}$$

where Rel = reliability of EBVs; PEV = prediction error variance of EBVs; and σ^2^_A_ = additive genetic variance of the trait.

Pairwise correlations between the sire EBVs in the two countries were subsequently calculated for the traits studied. These correlations were adjusted for reliability according to the method of Calo et al. [28] to derive an approximate estimate of the genetic correlation between traits in the two countries as follows:

(3) $$r_{g}=\frac{\sqrt{\sum_{i=1}^{n}\;{\rho_{i}}_{,\mathrm{EBV}}\times \sum_{i=1}^{n}\;{{\rho_{i}}_{,\mathrm{EBV}}}^{\prime }}}{\sum_{i=1}^{n}\;{\rho_{i}}_{,\mathrm{EBV}}\times {{\rho_{i}}_{,\mathrm{EBV}}}^{\prime }}\times {r_{\mathrm{EBV},\mathrm{EBV}}}^{\prime }$$

where r_g_ = approximate genetic correlation between two countries; ρ_i,EBV_ = the reliability of the EBV in country i; ρ_i,EBV’_ = the reliability of the EBV in country j; and r_EBV,EBV’_ = Pearson correlation between the EBVs from the two countries.

Standard error for the above approximate genetic correlations was calculated using the following formula proposed by Onyiro et al. [29]:

(4) $$\mathrm{SE}=\sqrt{\frac{1-r_{g}^{2}}{n-2}}$$

where SE = standard error of approximate genetic correlation between two countries; r_g_ = approximate genetic correlation between two counties; and n = number of common sires with EBVs in both countries.

Subsequently, joint bivariate analyses were performed on the combined datasets from the two countries, using model (1) above. The objective here was to derive the animal EBVs of all animals on the scale of each of the two countries. This analysis used the same variance component estimates previously derived in the within-country analyses. Genetic covariance estimates were derived from the approximate genetic correlations described above, as follows:

(5) $$\mathrm{Co}v_{1,2}={\;r}_{g}\times (\mathrm{st}g_{1}\times \mathrm{st}g_{2})$$

where Cov_1,2_ = genetic covariance of two traits; stg_1_ = genetic standard deviations for one trait; and stg_2_ = genetic standard deviations for the other trait.

Residual covariance between the two countries was set to zero since each animal had a record in only one of the two countries.

EBVs of sires from these bivariate analyses pertained to all sires in France and all sires in Greece who had progeny with records in at least one country. These EBVs were expressed on the scale of each country. The Pearson and Spearman rank correlations between sire EBVs in the two country scales were calculated.

#### 2.4.3. Predicted Response to Within- and Across-Country Genetic Selections

A deterministic simulation was performed to derive the predicted genetic gain (response to selection) for each trait and country from sire selection within and across countries using the following formula [30]:

(6) $$\Delta G=i\;\times r\;\times \sigma_{A}$$

where ΔG = predicted genetic gain per generation; i = selection intensity; r = accuracy of selection; and σ_A_ = additive genetic standard deviation for a specific trait.

A total of 30 and 600 sires were considered as selection candidates within Greece and France, respectively, according to the currently available numbers and the envisaged needs of each country. The number of sires in France reflects the needs of nucleus farms (ca. 400 flocks) [31]. Different selection intensities from the selection of the top 10 and 15 sires within and across-country were simulated. Accuracy of selection was the square root of sire EBV reliability (minimum threshold of 0.30) estimated in the previous analyses. Additive genetic standard deviation was the square root of the additive genetic variance for each trait and country from the above analyses.

## 3. Results

### 3.1. Descriptive Statistics

Descriptive statistics of the studied animal traits (phenotypes) in Greece and France are presented in Table 2. Mean milk yield was slightly higher in Greece, whereas fat and protein contents were higher in France. Mean lactose and SNF content in Greece were 5.08% and 12.09%, respectively.

### 3.2. Genetic Parameters

Estimates of heritability for each of the studied animal traits and genetic correlations between the two countries are presented in Table 3; repeatability estimates for traits in France are also reported. Similar statistically significant (*p* < 0.05) heritability estimates were reported for milk yield and fat content in Greece and France. Heritability of protein content was higher in France compared to Greece, where a low borderline non-significant (*p* > 0.05) estimate was found. Significant (*p* < 0.05) low heritability estimates were reported for lactose and SNF contents in Greece.

Approximate genetic correlations derived based on the correlation between the EBVs of common sires and grandsires, according to Calo et al. [28], are presented. Results showed a strong genetic correlation for milk yield and protein content, suggesting a very limited G × E interaction. A moderate to relatively high correlation was found for fat content, suggesting some degree of G × E interaction for this trait.

### 3.3. Sire Re-Ranking

Pearson and Spearman rank correlations between the EBVs of sires from Greece and France derived from the joint analysis are presented in Table 4. Strong correlations (>0.97) of EBVs were found for milk yield and protein content, consistent with the high genetic correlation between countries in Table 3. Correlations for fat content were lower, indicating possible sire re-ranking in the selection practices in the two countries.

### 3.4. Response to Selection

Predicted genetic gain for each trait and country based on selection of the top 10 and 15 sires within and across countries is presented in Table 5. Accuracy of EBVs ranged from 0.74 to 0.88. Selection intensity was for all traits higher when sires were selected based on the combined evaluations from the two countries compared to the evaluation in each country separately. As a result, response to selection was also higher in the case of combined evaluations with an increase in genetic gain between 0.1% and 68.2% depending on trait and country. The only exception was fat content in the case of France, where no increase in genetic gain was found. In both countries, the highest genetic gain was for protein content (59.0–68.2% for Greece and 0.0–0.2% for France) and the lowest for fat content (49.8–61.0% for Greece and 0.0% for France). For all studied traits, Greece was found to benefit more from combining genetic evaluations compared to France.

## 4. Discussion

The present study is the first to investigate the G × E interaction for milk production traits in purebred Lacaune sheep reared intensively in Greece and semi-extensively in France and estimate predicted response to selection based on genetic evaluations from both countries, compared to each country separately. Genetic correlations of the traits studied between animals raised in the two countries indicate no strong evidence of G × E interactions for milk yield and protein content. In the case of fat content, a moderately high genetic correlation was detected, suggesting that some degree of sire re-ranking could be expected. Overall, selection based on joint genetic evaluations from Greece and France is feasible, and results suggest an increase in genetic gain for both countries.

This study reported significant heritability estimates for all studied milk production traits of Lacaune sheep reared in Greece and France. The only exception was the heritability of protein content in Greece, which was not significantly different from zero. Our estimates for milk yield, fat content, and protein content of Lacaune sheep in France are consistent with previous literature [32,33]. To the best of our knowledge, however, this is the first study reporting heritability estimates for milk production traits of Lacaune sheep reared in Greece. Our results suggest similar estimates for milk yield and fat content of Lacaune sheep in Greece with the respective ones in France. However, substantial differences were found for protein content between the two countries. This could be attributed to the fact that Lacaune sheep in France have been consistently selected to improve milk protein content, whereas no such breeding practices have been applied in Greece. Moreover, they could be associated with differences in milk sampling and analyses between the two countries.

G × E interactions for the milk production traits of Lacaune sheep reared in Greece and France were investigated by estimating the respective genetic correlations. Environment was defined at the country level with further differences pertaining to farming systems (intensive in Greece and semi-extensive in France) and climatic conditions. Specifically, during the study period, the mean daily temperature and relative humidity in Greece were 14.4 °C (0.5–20.5 °C) and 63.7% (54.5–78.4%), whereas in France, these were 9.3 °C (0.6–20.5 °C) and 79.9% (47.2–93.4%), respectively, [34]. Bivariate analyses produced high standard errors, which could be related to the low connectedness between animals in the two countries (6 and 11 common sires and grandsires, respectively). This low connectedness is a result of the still limited use of AI in Greece [35]. To overcome this challenge, the method of Calo et al. (1973) was used to estimate the approximate genetic correlations for the studied traits based on the correlation between the EBVs of common sires and grandsires from univariate analyses.

Studies investigating G × E interactions for milk yield of sheep reared under different environments are limited. Garcia-Baccino et al. [19] reported a genetic correlation of 0.70 for milk yield of Latxa and Manech ewes between Spain and France. This correlation is lower compared to the one reported in our study concerning Lacaune ewes in Greece and France. However, our results are in general agreement with the genetic correlations (0.84–0.92) reported by Sanna et al. [18] for milk yield of Sarda ewes between low-to-medium and medium-to-high yield environments. Moreover, similar genetic correlations (0.80–0.90) have been reported for milk yield of Holstein [8,9,10] and Guernsey dairy cows [36] between different countries in Europe, America, and Australia, and Brown Swiss cattle reared under different altitudes in Germany [16].

In terms of milk quality, to the best of our knowledge, the present study is the first to investigate G × E interactions in sheep. Genetic correlations for fat and protein content in our study were lower compared to the ones obtained (>0.93) in Holstein cows between conventional and organic farming systems [11,16], different altitudes [16], and different levels of temperature–humidity index [15,37]. Such discrepancies could be related to differences in the connectedness of populations and environmental conditions between studies.

According to Mulder et al. [38,39], genetic correlations above 0.70 indicate limited or no G × E interactions between the traits studied in different countries, and hence, a higher genetic merit can be achieved with selection across compared to within countries. In our study, genetic correlations for milk yield and protein content of Lacaune sheep between the two countries were above 0.80, suggesting that Greece and France would benefit from the joint selection for these traits. Moreover, such results indicate that the animals selected by the French Lacaune breeding organization scheme are adapted to a diversity of environments. However, the correlation for fat content was approximately 0.60, suggesting some extent of G × E interaction. This interaction could be related to differences in the way of sampling and analyzing the milk between the two countries; all samples in France were collected during morning milking, whereas samples in Greece were alternately collected during morning or evening milking. Moreover, they could be related to differences in farming systems and feeding practices. Specifically, in Greece, the studied Lacaune sheep were reared intensively with high amounts of concentrates, whereas in France, the animals were reared semi-extensively, meeting their needs though forages (hay and silage) and grazing pastures. Additionally, differences in climatic conditions between Greece and France could also potentially explain the reported G × E interaction. The latter is in agreement with previous studies in dairy cows that reported G × E interactions for fat yield under different temperature–humidity index conditions [16,37].

The G × E interaction for fat content reported herein indicates that some degree of sire re-ranking might be expected. This is further supported by the Pearson and Spearman rank correlations between the EBVs of sires produced from the joint analysis and expressed in the scale of each country. Specifically, reported correlations of 0.80–0.84 for fat content confirm the re-ranking of sires. On the contrary, no such re-ranking was evident in the case of milk yield and protein content based on the respective correlations (0.97–0.99). Therefore, to deal with G × E interactions for fat content and avoid sire re-ranking, selection on combined data should be according to the scale in each country [39].

Based on all the above, a joint genetic evaluation and selection for milk production traits of Lacaune sheep in Greece and France is feasible. According to previous research in dairy cows and meat sheep, this approach provides a greater number of selection candidates, therefore increasing selection intensity and genetic gain [4,5,39,40]. This was also confirmed in our study, with selection of sires based on combined genetic evaluations from Greece and France resulting in a higher genetic gain for all traits in both countries. The only exception was fat content in France, where an equal genetic gain was found from analyses within and across countries. Moreover, our results suggest that a joint selection would be more beneficial for the Lacaune population in Greece compared to France for all traits (genetic gain up to 68.2% and 0.2%, respectively). The latter is attributed to the lower number of sires with available genetic evaluations in Greece in relation to France, which increases selection intensity [4].

A certain degree of caution should be exercised due to the low connectedness of populations in the present study and results should be re-examined when a higher number of common sires between Greece and France is achieved. Moreover, accuracy of selection in this study was derived from the EBV reliability of sires used for the G × E analyses, and therefore, it is probably underestimated. Finally, although both countries would benefit from a joint genetic evaluation and selection for the studied traits, breeding strategies should be tailored to the needs and conditions in each country, subject to accurate and systematic recording of phenotypes. To accommodate these strategies and account for the practical implementation of sire selection, future research should investigate genetic gains after combining EBVs for milk yield and composition in overall selection indices.

## 5. Conclusions

In the present study, G × E interactions for milk yield and composition of Lacaune sheep reared intensively in Greece and semi-extensively in France and response to selection based on combined genetic evaluations from the two countries were investigated. Genetic correlations suggest no or limited G × E interactions for milk yield and protein content; however, interactions and subsequent re-ranking of sires were found for fat content. Results suggest the feasibility of genetic evaluation and selection for milk production traits of Lacaune sheep using combined data from Greece and France. The two populations would benefit more through the selection of sires according to the scale in each country, especially in the case of fat content. Joint genetic evaluation and selection are expected to increase selection intensity and genetic gain for the studied traits in both countries.

## Figures and Tables

**Table 1:** Description of datasets from Greece and France.

Characteristic	Greece	France
Study period	2021–2022	2019–2022
Number of ewes	1658	4859
Number of sires	14	64
Number of records	1658	7166
Type of records	Single	Multiple
Number of first parity records	858	4047
Number of second parity records	800	2564
Number of third and above parity records	0	555
Milking period length in days (SD ^1^)	168.7 (17.72)	168.8 (38.94)
Age at lambing in months (SD ^1^)	20.4 (6.00)	20.5 (10.65)
Days from lambing to first sampling (SD ^1^)	34.2 (6.66)	51.5 (11.82)

^1^ SD = standard deviation.

**Table 2:** Descriptive statistics of milk production traits per country.

Phenotype	Country	N ^1^	Mean	SD ^2^	Min ^3^	Max ^4^
Milk yield (kg/ewe/milking period)	Greece	1658	362.58	149.62	37.9	865.66
	France	7164	329.96	98.48	13.6	836.00
Fat content (%) *	Greece	1538	6.16	0.81	3.93	9.37
	France	7145	6.77	0.92	2.87	10.00
Protein content (%) *	Greece	1566	4.48	0.48	2.94	6.54
	France	7166	5.52	0.52	3.97	8.04
Lactose content (%)	Greece	1615	5.08	0.20	3.19	6.65
SNF ^5^ content (%)	Greece	1568	12.09	0.60	10.09	14.73

^1^ N = number; ^2^ SD = standard deviation; ^3^ Min = minimum; ^4^ Max = maximum; ^5^ SNF = solids-non-fat content. * Fat and protein contents for all animals in France were estimated from samples collected during morning milking.

**Table 3:** Heritability (h^2^) and repeatability (r) within country and genetic correlations between Greece and France for the studied animal traits (standard errors in parenthesis).

Trait	Country	h^2^	r	Genetic Correlations
Milk yield (kg/ewe/milking period)	Greece	0.19 (0.09) *		0.86 (0.13) *
	France	0.24 (0.05) *	0.41 (0.02) *	
Fat content (%)	Greece	0.30 (0.13) *		0.59 (0.21) *
	France	0.34 (0.05) *	0.40 (0.02) *	
Protein content (%)	Greece	0.19 (0.10)		0.88 (0.12) *
	France	0.52 (0.02) *	0.52 (0.02) *	
Lactose content (%)	Greece	0.10 (0.05) *		
SNF ^1^ content (%)	Greece	0.14 (0.07) *		

^1^ SNF = solids-non-fat content. * Indicates statistically significant estimates (*p* < 0.05).

**Table 4:** Pearson and Spearman rank correlations between the estimated breeding values of sires from Greece and France for milk yield, fat content, and protein content based on the analysis across countries.

Trait	Pearson Correlation	Spearman Correlation
Milk yield (kg/ewe/milking period)	0.97	0.99
Fat content (%)	0.80	0.84
Protein content (%)	0.99	0.99

**Table 5:** Predicted response to top 10 and 15 sire selection based on genetic evaluations within and across countries for milk yield, fat content, and protein content.

Trait	Selection Scenario	Top Sires	N ^1^ Sires	PS% ^2^	i ^3^	r ^4^	σ_A_ ^5^	ΔG ^6^	ΔG% ^7^
Milk yield (kg/ewe/period)	Within—Greece	10	30	33.3	1.07	0.84	42.05	37.62	45.78
	Within—Greece	15	30	50.0	0.78	0.84	42.05	27.56	35.57
	Within—France	10	600	1.7	2.46	0.81	26.60	53.18	99.89
	Within—France	15	600	2.5	2.32	0.81	26.60	50.22	100
	Across—Greece	10	630	1.6	2.48	0.79	42.05	82.20	100
	Across—Greece	15	630	2.4	2.34	0.79	42.05	77.47	100
	Across—France	10	630	1.6	2.48	0.81	26.60	53.24	100
	Across—France	15	630	2.4	2.34	0.81	26.60	50.18	100
Fat content (%)	Within—Greece	10	30	33.3	1.07	0.88	0.41	0.38	50.16
	Within—Greece	15	30	50.0	0.78	0.88	0.41	0.28	38.98
	Within—France	10	600	1.7	2.46	0.84	0.46	0.95	100
	Within—France	15	600	2.5	2.32	0.84	0.46	0.90	100
	Across—Greece	10	630	1.6	2.48	0.75	0.41	0.76	100
	Across—Greece	15	630	2.4	2.34	0.75	0.41	0.72	100
	Across—France	10	630	1.6	2.48	0.79	0.46	0.91	100
	Across—France	15	630	2.4	2.34	0.79	0.46	0.86	100
Protein content (%)	Within—Greece	10	30	33.3	1.07	0.74	0.05	0.04	40.96
	Within—Greece	15	30	50.0	0.78	0.74	0.05	0.03	31.83
	Within—France	10	600	1.7	2.46	0.84	0.30	0.63	99.83
	Within—France	15	600	2.5	2.32	0.84	0.30	0.59	100
	Across—Greece	10	630	1.6	2.48	0.77	0.05	0.09	100
	Across—Greece	15	630	2.4	2.34	0.77	0.05	0.08	100
	Across—France	10	630	1.6	2.48	0.84	0.30	0.63	100
	Across—France	15	630	2.4	2.34	0.84	0.30	0.59	100

^1^ N = number; ^2^ PS% = proportion selected; ^3^ i = selection intensity; ^4^ r = accuracy of selection candidate EBVs; ^5^ σ_A_ = genetic standard deviation of trait; ^6^ ΔG = response to selection (genetic gain); ^7^ ΔG% = percentage of genetic gain from genetic evaluation within compared to across countries.

## Data Availability

The data presented in this study are contained within the article and Supplementary Material (Dataset S1 and Dataset S2).

## Supplementary Materials

The following supporting information can be downloaded at: https://www.mdpi.com/article/10.3390/vetsci12030194/s1, Dataset S1: Combined pedigree file of French and Greek Lacaune sheep; Dataset S2: Total dataset used for the analyses; Figure S1: Relationship (box and whisker plots) of milk production traits with studied farms in Greece; Figure S2: Relationship (box and whisker plots) of milk production traits with studied years in Greece; Figure S3: Relationship (box and whisker plots) of milk production traits with parity of studied Lacaune ewes in Greece; Figure S4: Relationship (scatterplots) of milk production traits with milking period length of studied Lacaune ewes in Greece; Figure S5: Relationship (scatterplots) of milk production traits with age at lambing of studied Lacaune ewes in Greece; Figure S6: Relationship (scatterplots) of milk production traits with days from lambing to first sampling of studied Lacaune ewes in Greece; Figure S7: Relationship (box and whisker plots) of milk production traits with studied years in France; Figure S8: Relationship (box and whisker plots) of milk production traits with parity of studied Lacaune ewes in France; Figure S9: Relationship (scatterplots) of milk production traits with milking period length of studied Lacaune ewes in France; Figure S10: Relationship (scatterplots) of milk production traits with age at lambing of studied Lacaune ewes in France; Figure S11: Relationship (scatterplots) of milk production traits with days from lambing to first sampling of studied Lacaune ewes in France; Figure S12: Relationship (box and whisker plots) of milk production traits with number of lambs born from studied Lacaune ewes in France.

## Abbreviations

The following abbreviations are used in this manuscript:

AI	Artificial insemination
EBV	Estimated breeding value
G × E	Genotype-by-environment
i	Selection intensity
ICAR	International Committee of Animal Recording
Min	Minimum
Max	Maximum
N	Number
PEV	Prediction error of variance
PS	Proportion selected
r	Accuracy of selection
SD	Standard deviation
SE	Standard error
SNF	Solids-non-fat
ΔG	Predicted genetic gain
σA	Additive genetic standard deviation
