# Supplementary material for: Genotype-by-Environment Interactions and Response to Selection for Milk Production Traits in Lacaune Sheep from Greece and France
Source: Vet Sci. 2025 Feb 21;12(3):194. doi: 10.3390/vetsci12030194 (PMC11946213; doi:10.3390/vetsci12030194)
Supplement: Supplementary file 1 [file vetsci-12-00194-s001.zip › Supplement S1.pdf]

Genotype by environment interactions and response to selection for milk production traits in Lacaune sheep from Greece and France

Sotiria Vouraki, Jean-Michel Astruc, Gilles Lagriffoul, Rachel Rupp, Georgios Banos, Georgios Arsenos

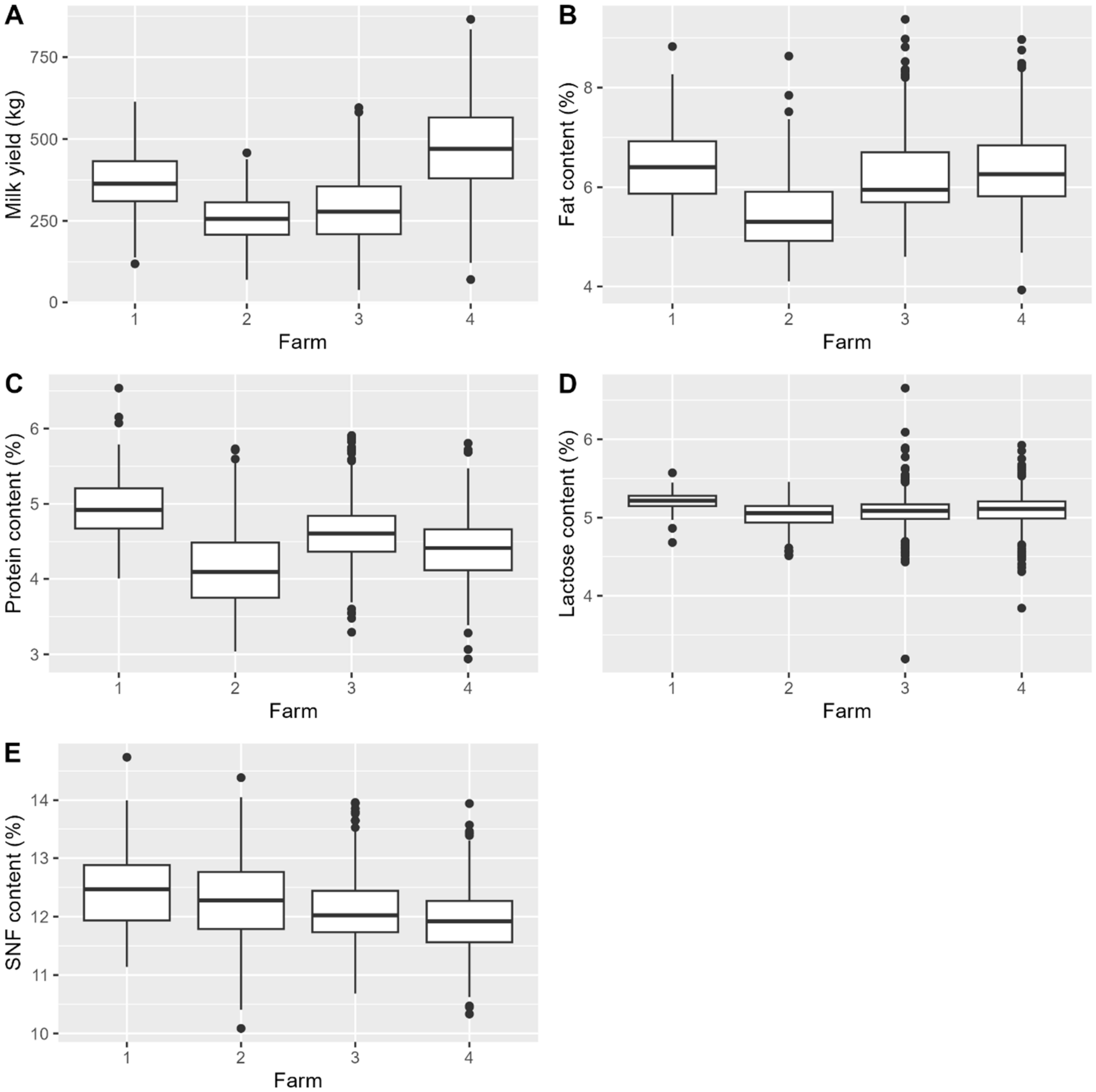

Figure S1. Relationship (box and whisker plots) of milk production traits with studied farms in Greece.

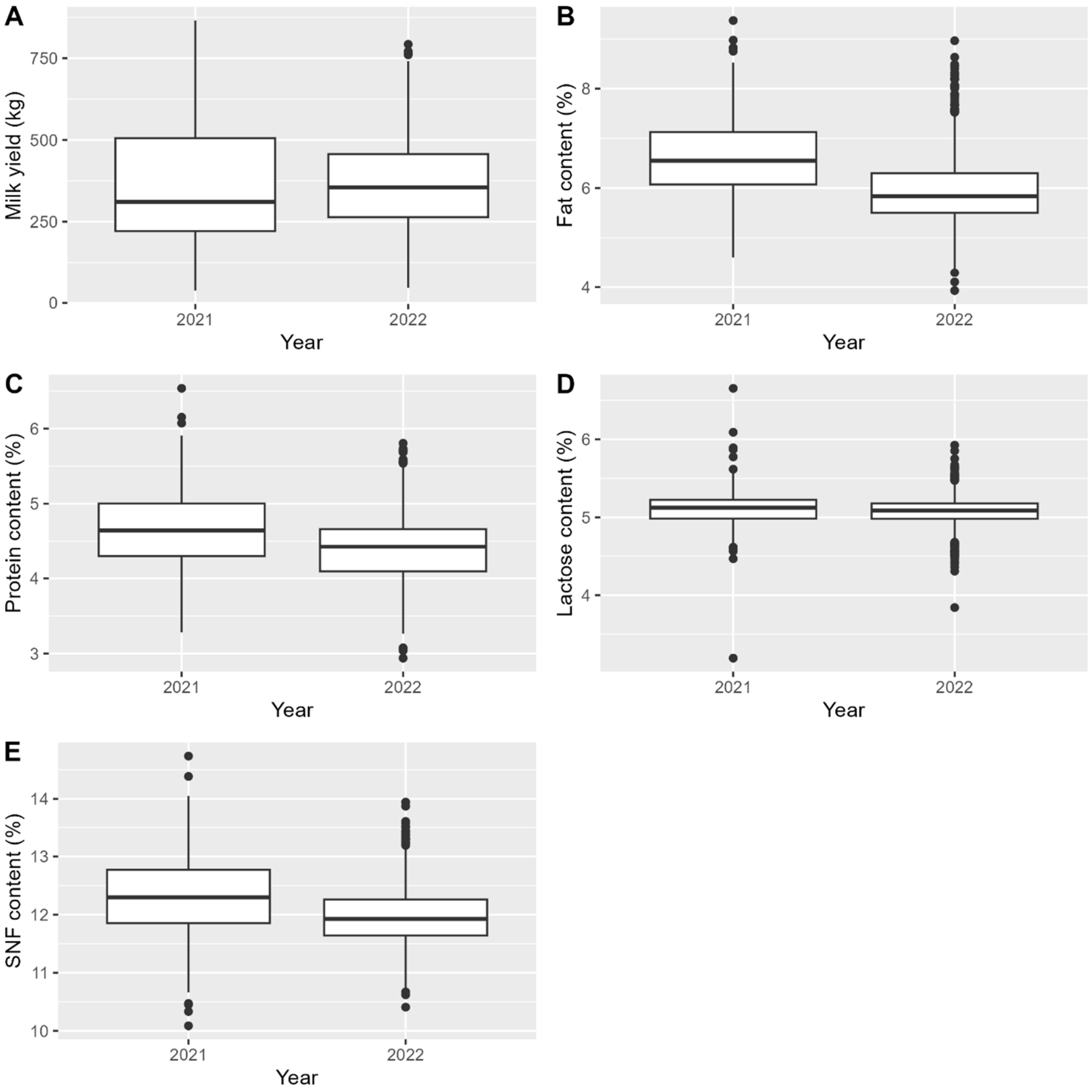

Figure S2. Relationship (box and whisker plots) of milk production traits with studied years in Greece.

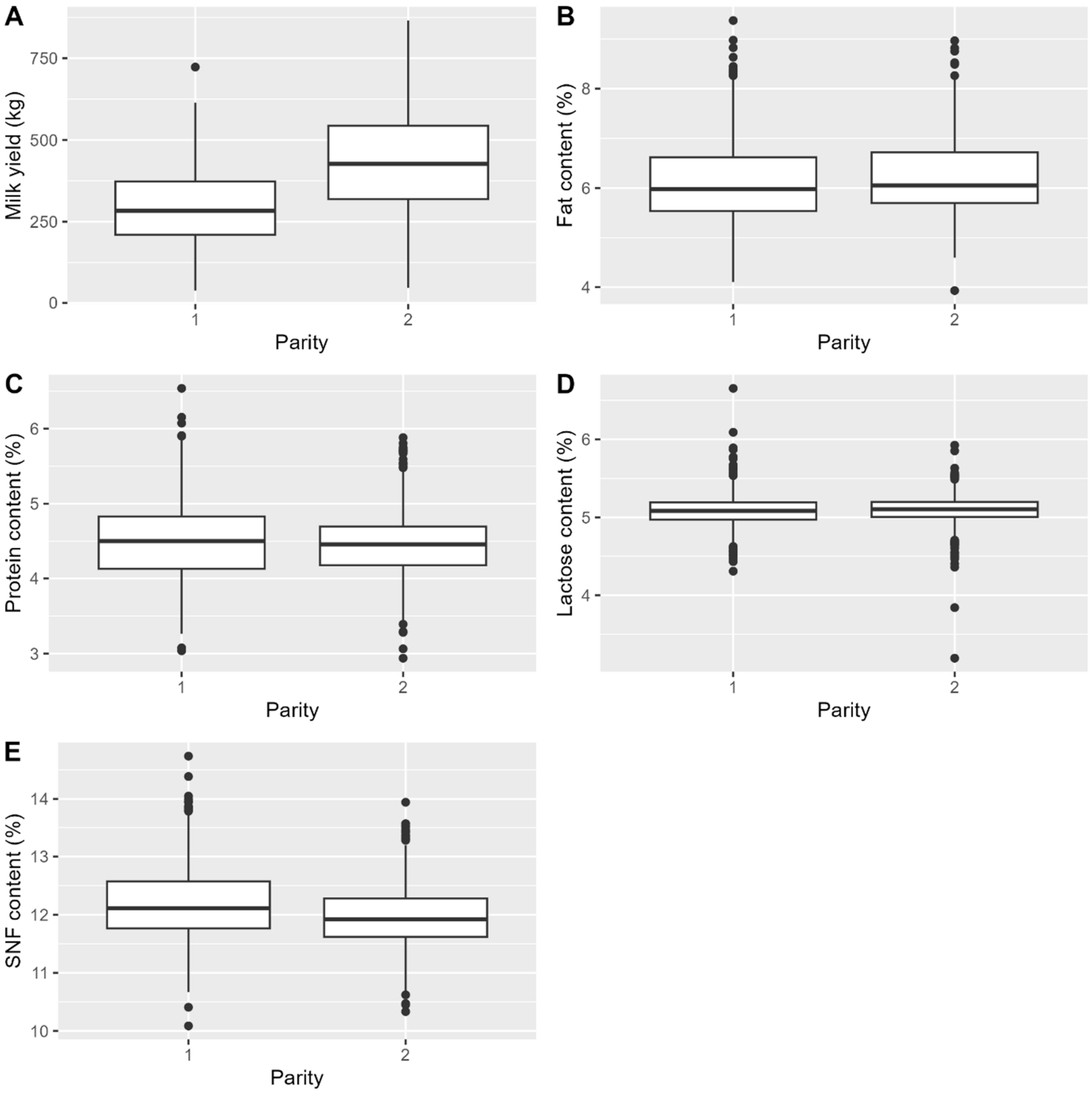

Figure S3. Relationship (box and whisker plots) of milk production traits with parity of studied Lacaune ewes in Greece.

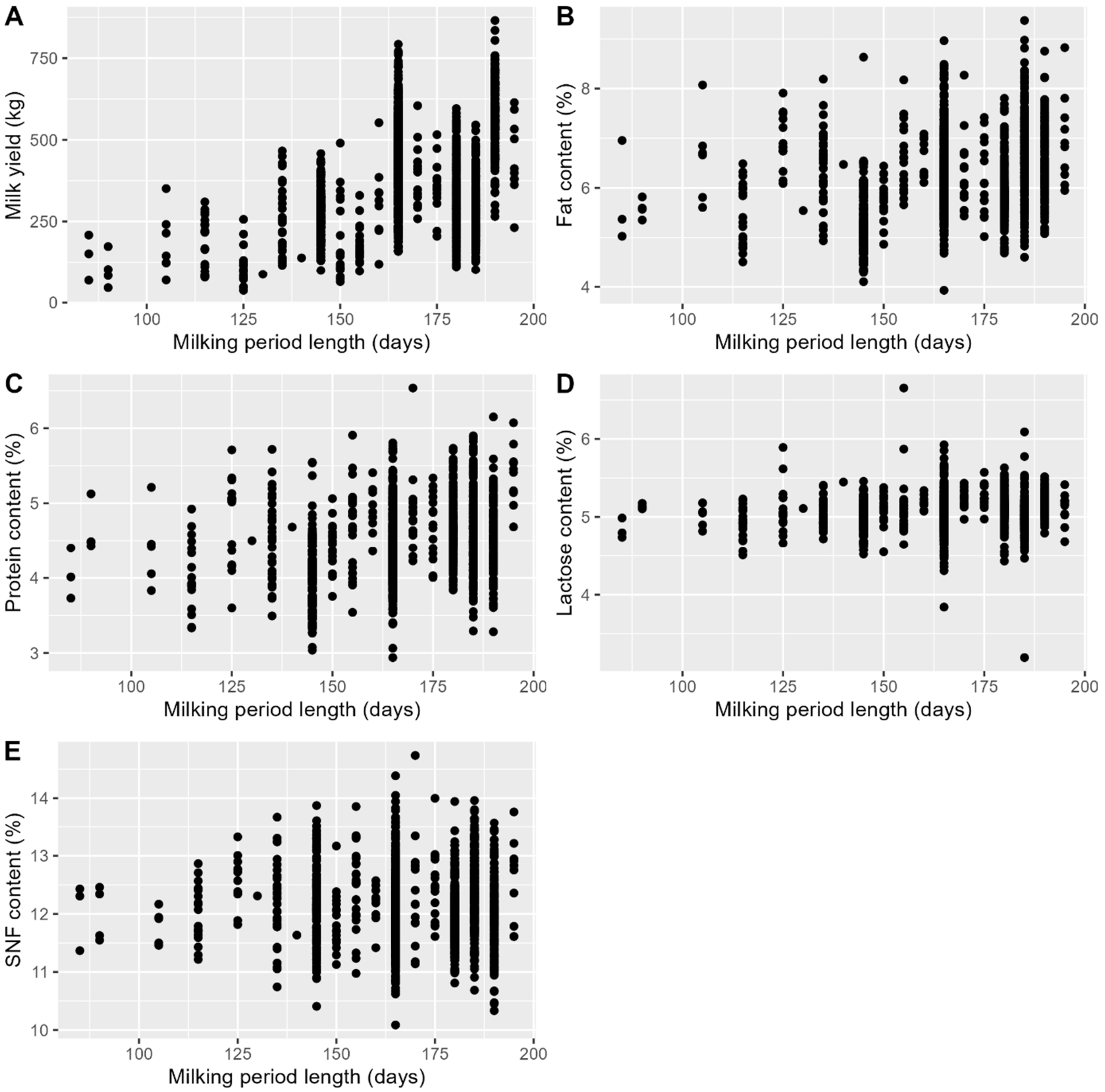

Figure S4. Relationship (scatterplots) of milk production traits with milking period length of studied Lacaune ewes in Greece.

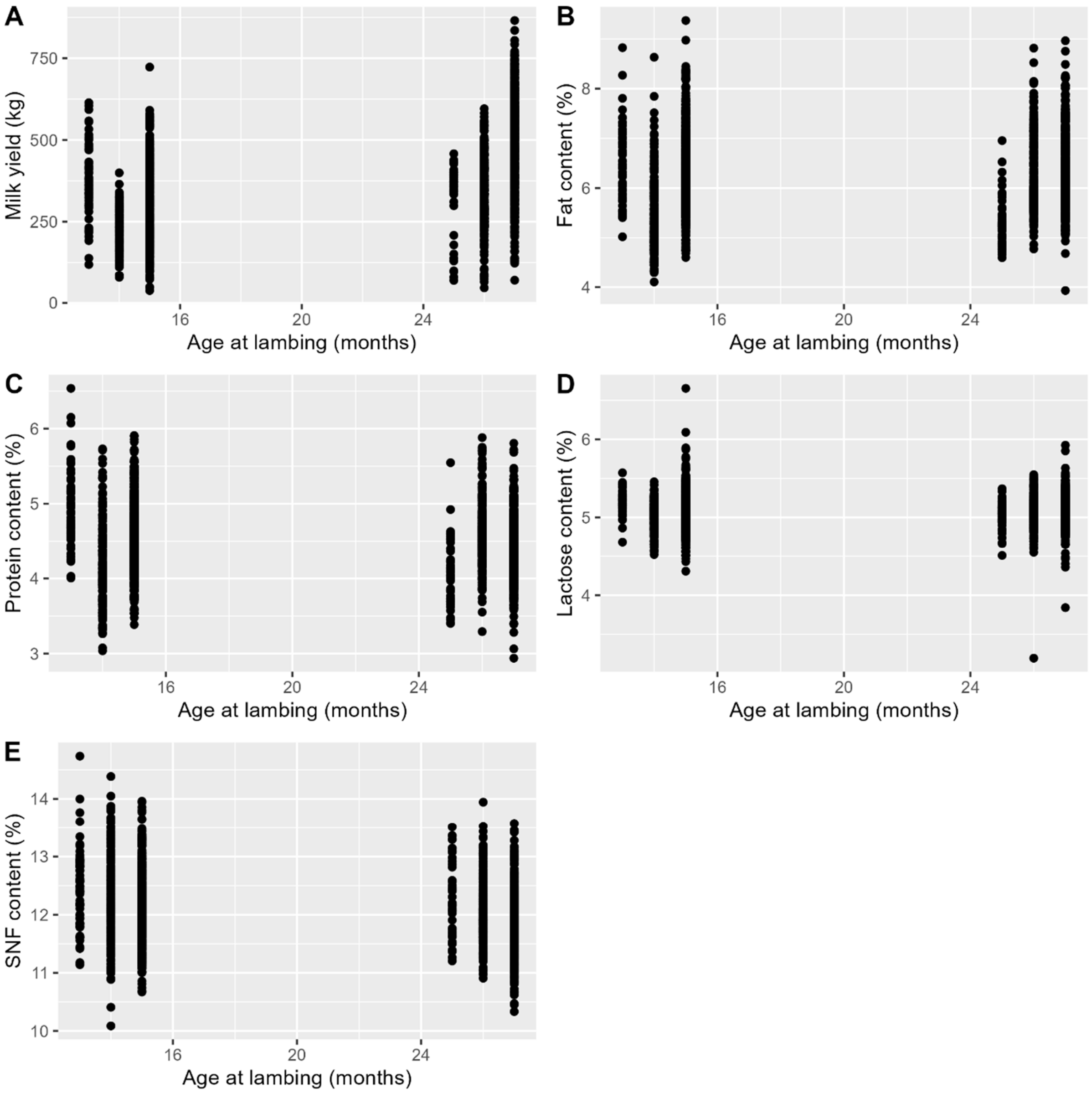

Figure S5. Relationship (scatterplots) of milk production traits with age at lambing of studied Lacaune ewes in Greece.

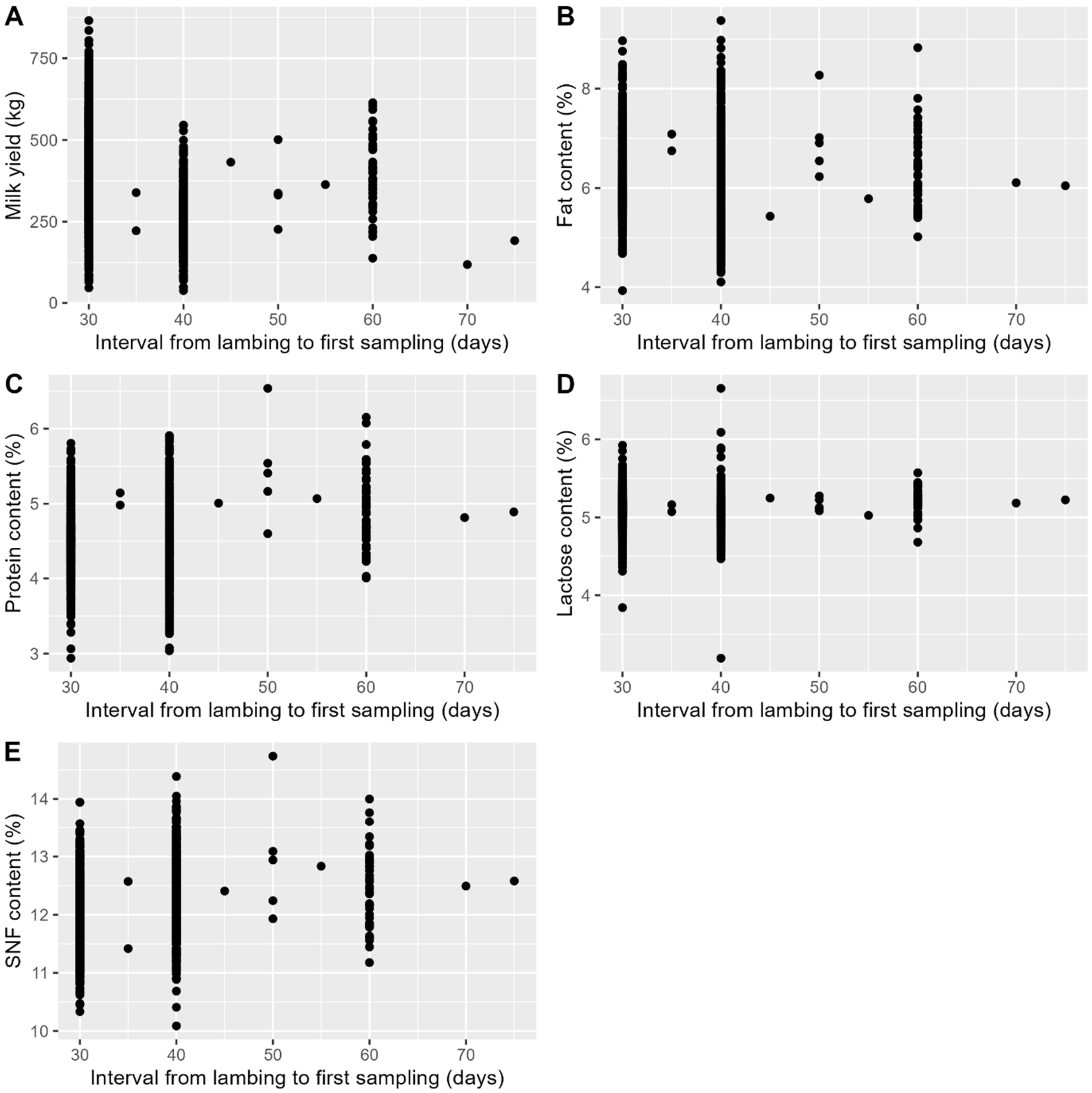

Figure S6. Relationship (scatterplots) of milk production traits with days from lambing to first sampling of studied Lacaune ewes in Greece.

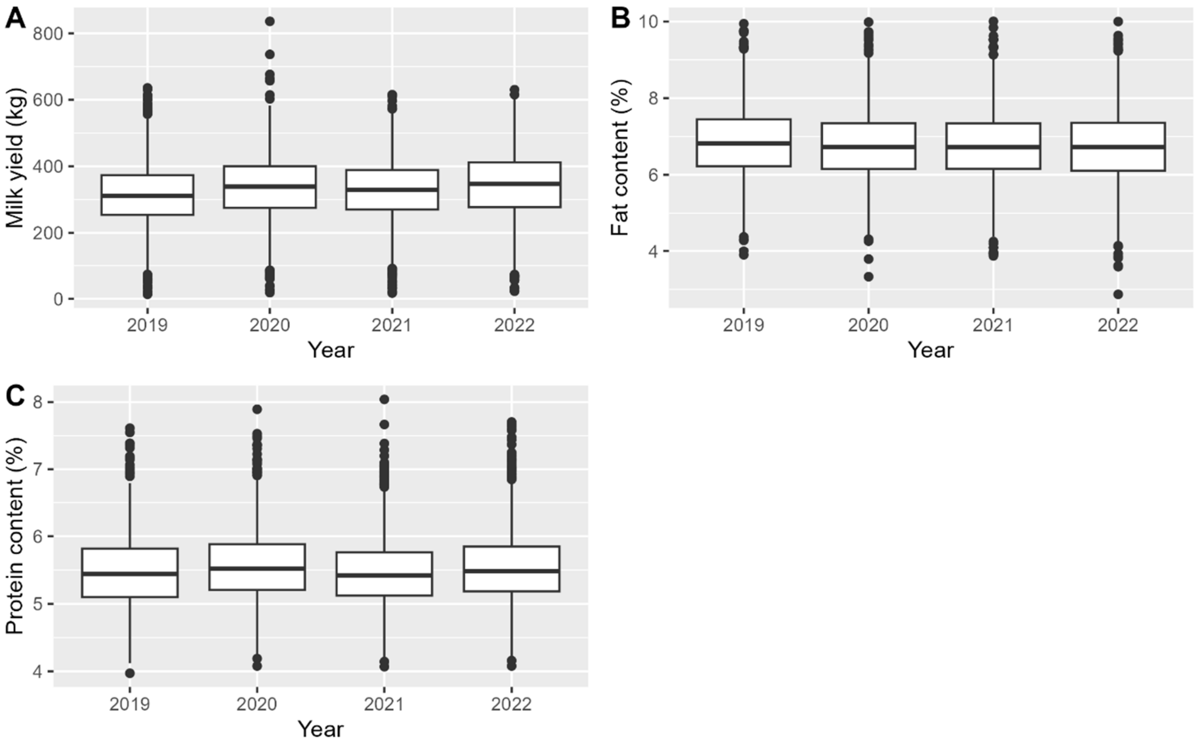

Figure S7. Relationship (box and whisker plots) of milk production traits with studied years in France.

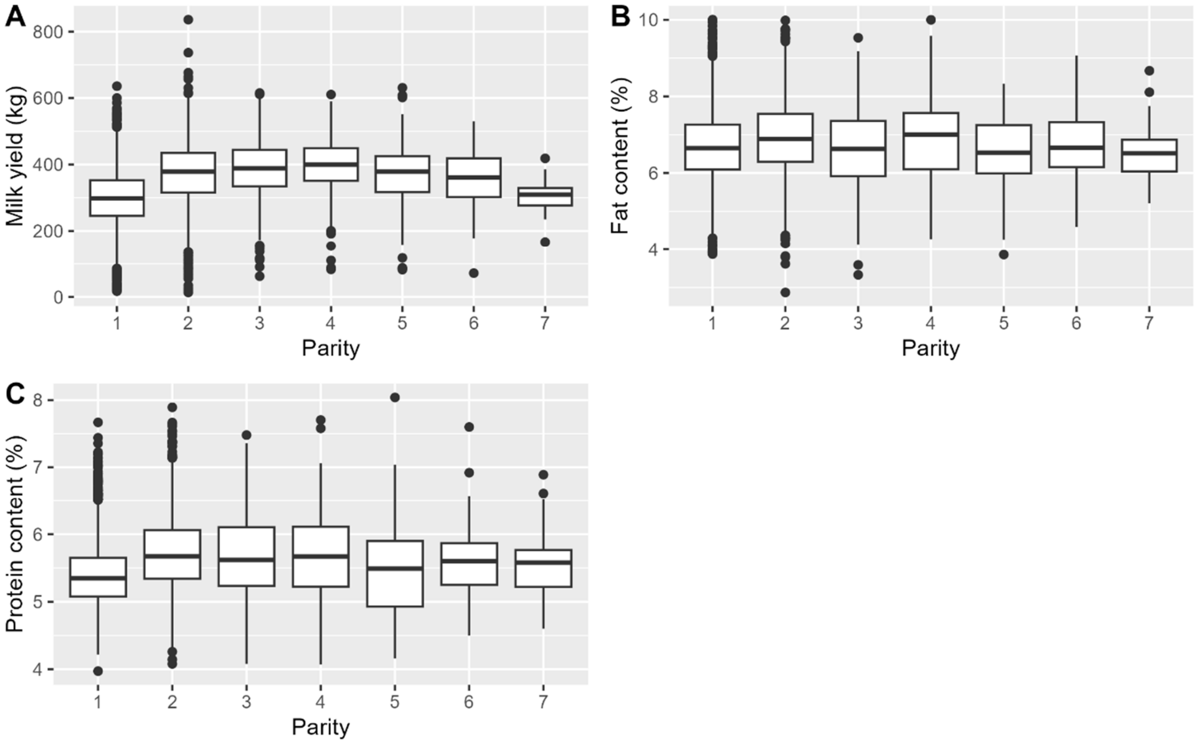

Figure S8. Relationship (box and whisker plots) of milk production traits with parity of studied Lacaune ewes in France.

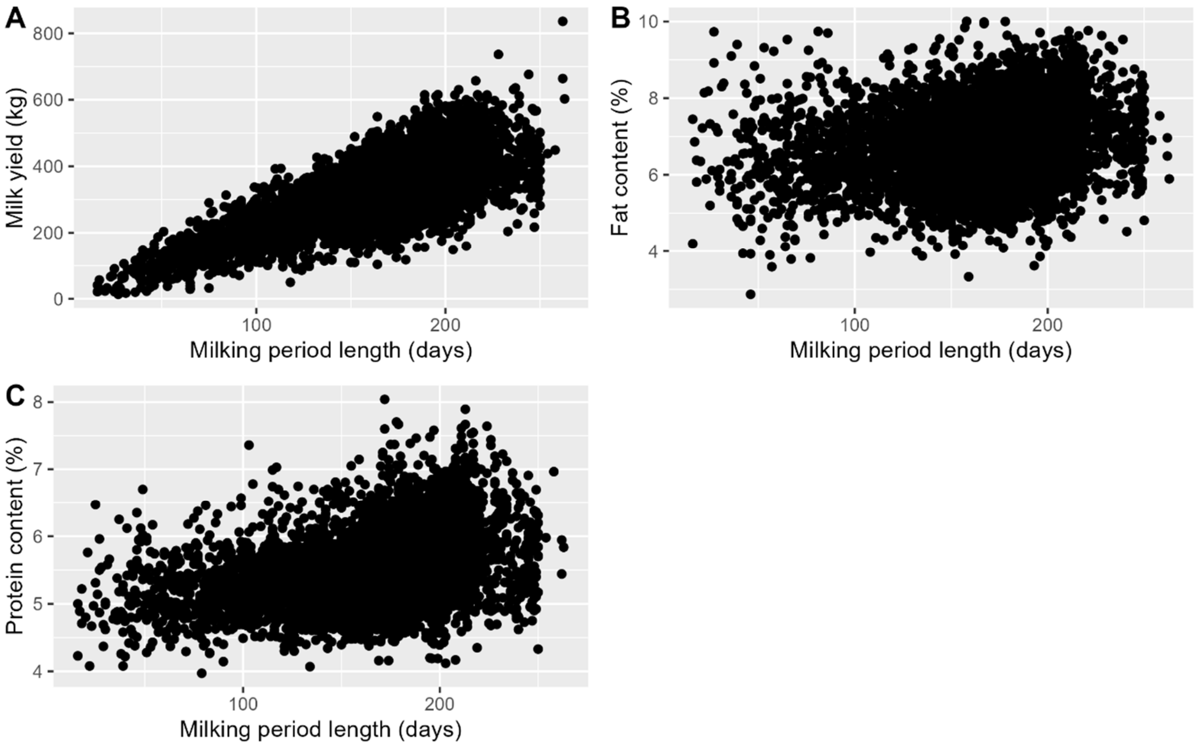

Figure S9. Relationship (scatterplots) of milk production traits with milking period length of studied Lacaune ewes in France.

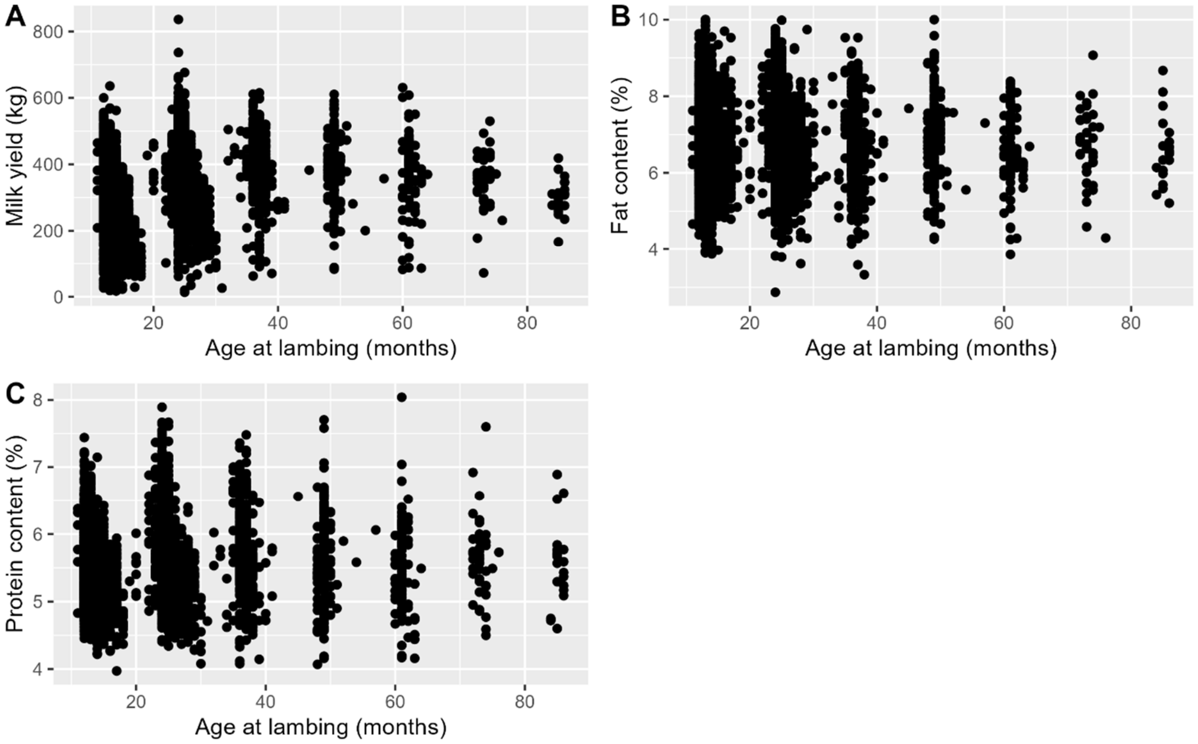

Figure S10. Relationship (scatterplots) of milk production traits with age at lambing of studied Lacaune ewes in France.

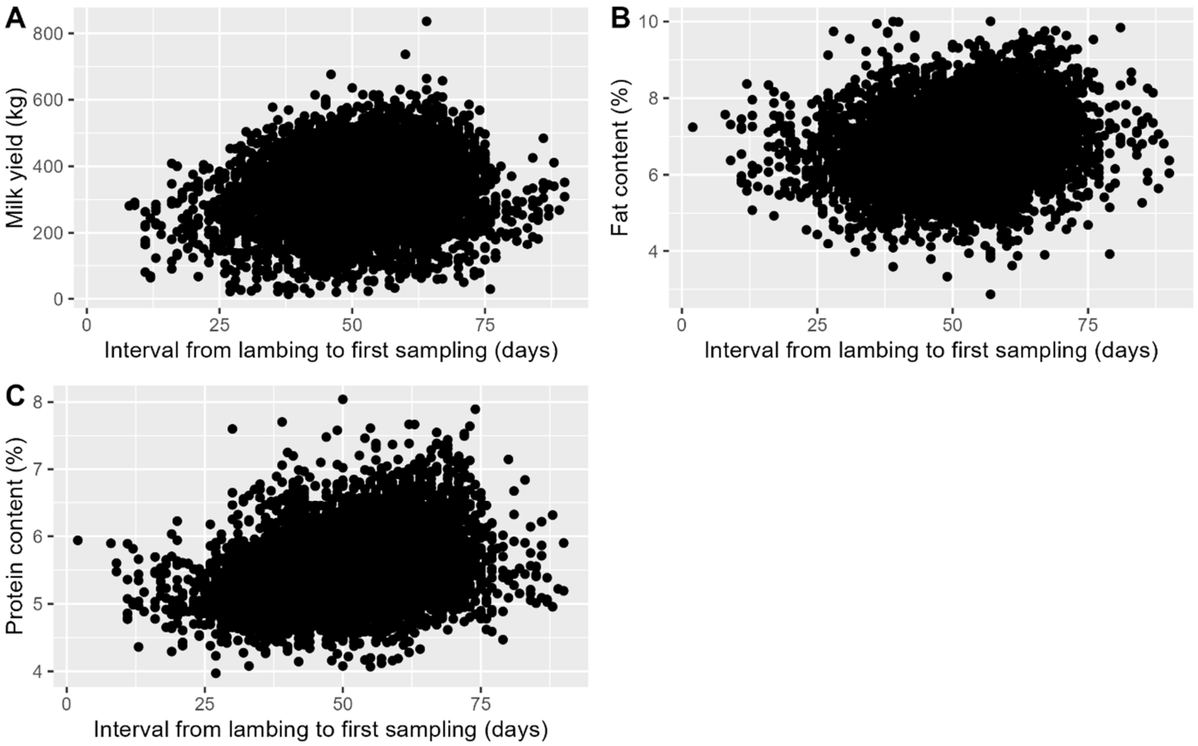

Figure S11. Relationship (scatterplots) of milk production traits with days from lambing to first sampling of studied Lacaune ewes in France.

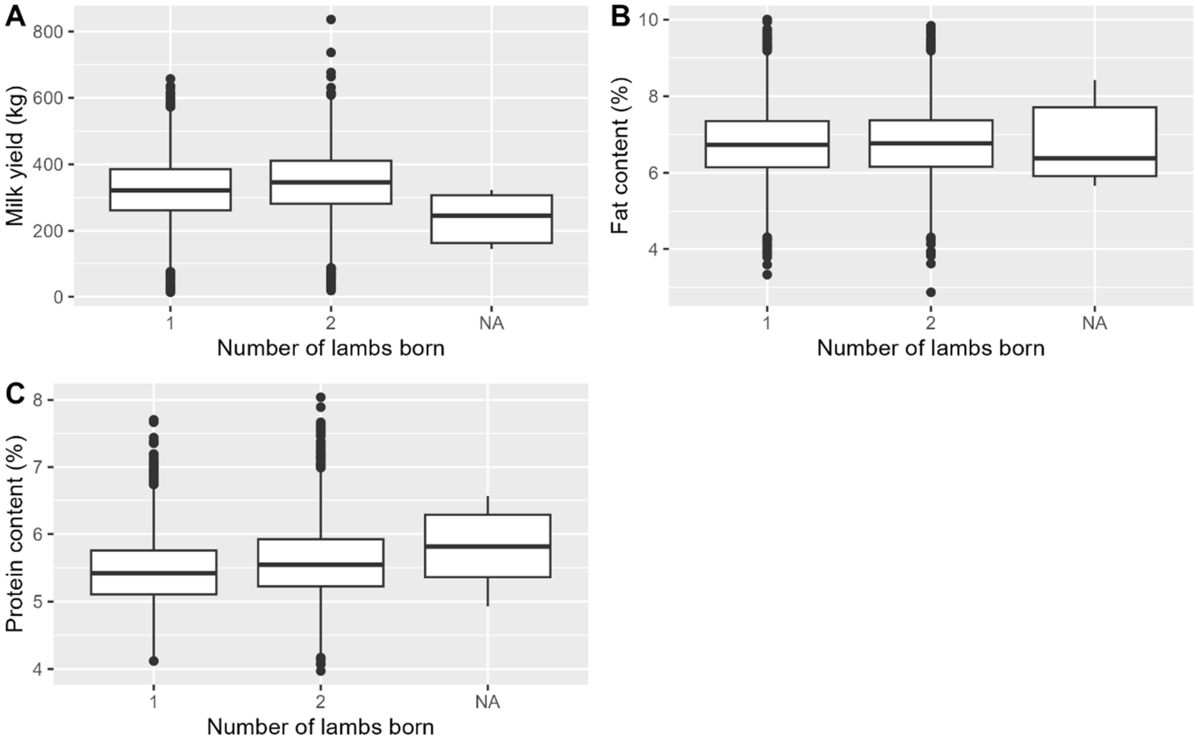

Figure S12. Relationship (box and whisker plots) of milk production traits with number of lambs born from studied Lacaune ewes in France.
